# The relation between, metabolic syndrome and quality of life in patients with Systemic Lupus Erythematosus

**DOI:** 10.1371/journal.pone.0187645

**Published:** 2017-11-07

**Authors:** Domenico Paolo Emanuele Margiotta, Fabio Basta, Giulio Dolcini, Veronica Batani, Luca Navarini, Antonella Afeltra

**Affiliations:** Unit of Allergology, Clinical Immunology and Rheumatology, Università Campus Bio-Medico di Roma, Rome, Italy; National Institute of Health, ITALY

## Abstract

**Introduction:**

Systemic Lupus Erythematosus (SLE) is associated to an increased prevalence of Metabolic Syndrome (MeS) and to a reduction of Quality of Life (QoL). The aim of this study is to evaluate the association between MeS and QoL in SLE.

**Methods:**

SLE patients were consecutively enrolled in a cross sectional study. MeS was defined according to IFD definition. Therapy with glucocorticoids (GC) and antimalarial was analyzed as cumulative years of exposure. We used a cut off of 7.5 mg of prednisone to define high daily dose of GC. QoL was quantified using SF-36. We used BDI and HAM-H to assess symptoms of mood disorders. Fatigue was evaluated using Facit-Fatigue, physical activity using IPAQ, sleep quality using PSQI and alexithymia using TAS-20.

**Results:**

We enrolled 100 SLE patients. MeS prevalence was 34%. Patients with MeS presented reduced scores in SF-36 MCS and PCS compared to patients without MeS (p 0.03 and p 0.004). BDI and HAM-H score were significantly higher in patients meeting MeS criteria compared to subjects without MeS (p 0.004, p 0.02). These results were confirmed after adjustment for confounders. Compared to patients without MeS, those with MeS presented higher age, lower education level, higher recent SELENA-SLEDAI, higher number of flares, increased SDI, longer cumulative exposure to high dose GC and shorter duration of antimalarial therapy. In the multiple logistic regression model, the variable associated to the Odds Ratio of having MeS were: the average of recent SELENA-SLEDAI (OR 1.15 p 0.04), the years of exposure to high dose of GC (OR 1.18 p 0.004), the years of exposure to antimalarials (OR 0.82 p 0.03) and the BDI score (OR 1.1 p 0.005).

**Conclusion:**

A modern management of SLE should not miss to take all the possible measures to ensure an adequate QoL to SLE patients, with particular attention to those affected by MeS.

## Introduction

Metabolic Syndrome (MeS) is a cluster of cardiovascular diseases (CVDs) risk factors in which insulin-resistance (IR) and visceral adiposity play a pivotal role. In the last decade, several definitions of MeS have been proposed. The most widely used MeS definitions are the National Cholesterol Education Program (NCEP) Adult Treatment Panel III (ATP III) [1] and the International Diabetes Federation (IDF) ones [2].

All definitions include a measure of visceral obesity such as waist circumference, measures of dyslipidemia including raised triglycerides and low high-density lipoprotein (HDL) cholesterol, measures of IR usually expressed by fasting plasma glucose (FPG) and arterial hypertension.

Epidemiological studies clearly demonstrated that MeS is not just the sum of CVDs risk factors, but is an independent CVDs risk factor [3]. A recent meta-analysis including nearly 1 million individuals showed that the MetS was associated with almost twice the relative risk of CVDs prevalence and mortality and a 1.6 relative risk of all-cause mortality [4]. In addition, people with metabolic syndrome have a fivefold greater risk of developing type 2 diabetes [5]. The clustering of CVD risk factors that typifies the metabolic syndrome is now considered to be the driving force for a CVD epidemic.

In general population, a poor Health Related Quality of Life (HRQoL), especially in physical domain, has been described in patients with MeS [6–10]. Ford et al. compared HRQoL in patients with and without MeS in a cross-sectional analysis of 1859 subjects from the National Health and Nutrition Examination Survey, demonstrating that patients with the metabolic syndrome were more likely to have fair or poor health, mentally unhealthy days and activity limitation days [7].

Moreover, MeS seems to be associated to depression and chronic fatigue, both factors tightly connected to HRQoL [11, 12].

Systemic Lupus Erythematosus (SLE) is a systemic autoimmune disease with a possible involvement of all organs and systems, with an extremely wide spectrum of clinical manifestations. The immune-pathogenesis of SLE is complex and involves both innate and adaptive immunity. SLE therapy varies according to clinical manifestations and is based on the use of glucocorticoids, synthetic antimalarials, immunosuppressants and, recently, biological drugs (in particular, directed against B-lymphocytes) [13, 14]. Crescent literature data clearly demonstrated an increased CVDs prevalence and mortality in SLE, due to accelerated atherosclerosis [15–17]. SLE patients present higher prevalence of insulin-resistance and MeS compared to age and sex matched healthy controls [18–24]. According to available studies, in SLE patients, MeS seems to be related to traditional CVDs risk factors, age, disease duration, low complement levels, renal involvement and glucocorticoid therapy [20–24]. Data from the Systemic Lupus International Collaborating Clinics (SLICC) Registry for Atherosclerosis inception cohort, concerning 1494 recently diagnosed SLE, demonstrated that MeS was present at enrollment visit in 16% of patients suggesting that lupus-related inflammatory factors could facilitate insulin-resistance and MeS development. In the same study, factors associated to MeS in multivariate analysis were renal lupus, higher corticosteroid doses, Korean and Hispanic ethnicity [25]. In a longitudinal analysis of patients enrolled in SLICC registry, in addition to the factors already highlighted, presence of organ damage according to SLICC damage index and higher disease activity were independently associated with MeS over the first 2 years of follow-up [26].

Despite the improvement in SLE prognosis in the last decades, patients are still burdened by poor quality of life, often associated to pain, depression and fatigue, all common lupus manifestations [25].

The aim of this study was to compare QoL in SLE patients with and without MeS, after adjustment for possible confounders. Furthermore, secondary objective of this study was evaluate, in a multivariable model, if MeS could be a factor related to QoL in SLE.

## Methods

### Study population

Patients affected by SLE according to SLICC classification criteria [27] were consecutively enrolled at University Campus Bio-Medico outpatient clinic between January 2015 and December 2015. Patients were enrolled from the inception cohort of our Lupus Clinic. All patients enrolled were continuously followed in our Lupus Clinic from diagnosis until the enrollment in this study. The opportunity to take part in the study was orally proposed during outpatient outreach visits. Exclusion criteria for SLE patients were: recent pregnancy (<2 years before enrollment), active malignancy, end-stage lupus nephritis, treatment with Belimumab in the last two years, previous diagnosis of mood disorders or ongoing therapy for mood disorders.

### Sample size calculation

For sample size calculation and power analysis we considered data concerning QoL in general population with and without MeS [8]. Setting a significance level of 0.05 (alpha), power at 80% (beta), an Effect size of 0.9, according to data on general population, and a proportion of subject exposed at 0.3 (considering MeS prevalence in SLE), we estimated a total group size of 46 patients including 14 patients affected by MeS. Sample size calculation and power analysis were performed using SAS University Edition, SAS Institute Inc., SAS Campus Drive, Cary, North Carolina 27513, USA.

### Ethical considerations

Ethics committee of Università Campus Bio-Medico di Roma approved the study, which complied with the Declaration of Helsinki. All the study participants provided signed an informed consent prior to enrolment.

### Evaluation of metabolic parameters and CV risk factors

Clinical history, history of diabetes mellitus, dyslipidemia or hypertension, familiar or personal CVD history were assessed. Waist circumference, Waist/Hip ratio, body mass index (BMI), resting arterial blood pressure were recorded. In every patients enrolled fasting blood samples was analyzed for metabolic parameters as total cholesterol, low density lipoprotein (LDL) cholesterol, high density lipoprotein cholesterol (HDL) cholesterol, triglycerides, fasting glucose, C-reactive protein (CRP) using diagnostic commercial kits. Metabolic Syndrome was defined according to IFD criteria [2].

### Evaluation of SLE disease features, disease activity and damage accrual

Disease activity was defined using Safety of Estrogens in Lupus Erythematosus National Assessment disease activity index (SELENA-SLEDAI) [28, 29]. We calculated actual SELENA-SLEDAI and mean SELENA-SLEDAI of the last 12 months. Disease flares were assessed by SELENA-SLEDAI Flares Index (SFI) [30]. According to SFI, disease flares were classified in mild-moderate or severe. Disease damage was calculated using SLICC damage index (SDI) [31]. We evaluated the cumulative duration of exposure to glucocorticoids and to antimalarials, expressed in years. We used a cut off of 7.5 mg of prednisone or equivalents to define high daily dose glucocorticoid regimens, according to the evidences that link this cut off dose to an increased risk of damage accrual, in particular on cardio-metabolic damages [32]. According to this cut-off, we further evaluated the cumulative exposure to high dose glucocorticoids.

### Evaluation of QoL, mood disorders, fatigue, sleep quality, physical activity

To assess HRQoL we used Italian version of Medical Outcomes Study (MOS) 36-Items Short-Form Healthy Survey (SF-36) [33]. Fatigue was evaluated by Italian version of The Functional Assessment of Chronic Illness Therapy (FACIT)–Fatigue [34]. Depressive symptoms was quantified by the Beck Depression inventory (BDI version II) [35] and anxiety symptoms were evaluated by Hamilton Anxiety rating scale (HAM-H) [36]. Subjects were classified according to BDI cut-offs: BDI<13 no depression; BDI 14–19, mild depression; BDI 20–28 moderate depression; BDI 29–63 severe depression [37]. Physical activity was assessed by the International Physical Activity Questionnaire (IPAQ) and expressed according to categorical IPAQ total score: 1, patient inactive; 2 patient minimally active; 3 patient active according to Health Enhancing Physical Activity (HEPA) standards [38]. We also evaluated sleep quality by the Pittsburgh Sleep Quality Index (PSQI) [39]. Alexithymia construct was evaluated by Toronto Alexithymia Scale (TAS-20) [40].

### Statistical analysis

The two-tailed Student t-test or One-Way ANOVA was used to compare means when normal distribution was observed in a Kolmogorov-Smirnov test. When normality was not observed, the non-parametric Mann-Whitney test was used to compare medians. In order to take into account the impact on analyzed variable of possible confounders such as age, sex, education level and alexithymia, we compared the last square mean values (LS mean) of the analyzed variable in patients with and without MeS using a multiple mixed regression model (SAS PROC MIXED). The mixed models allowed us to compare LS means in an unbalanced design (the proportion of patients without MeS was significantly greater that subjects without MeS) and to compare variable with difference variance. SF-36 summary components MCS and PCS values were stratified in quartiles.

A generalized linear regression model (SAS PROC GENMOD) with multinomial distribution of dependent variable and cumulative logit link function was built to analyze the variables associated to MCS and PCS quartile (odds ratio to be in upper quartiles of MCS or PCS values vs lower one).

We used a generalized linear model (SAS PROC GENMOD) with binomial distribution of dependent variable (presence of MeS, yes or no) and logit link function to create a univariable and then a multivariable logistic regression model. Significance level adopted was two tailed p< 0.05. All statistical analyses were performed with SAS University Edition, SAS Institute Inc., SAS Campus Drive, Cary, North Carolina 27513, USA.

## Results

### SLE disease features

We enrolled 100 SLE patients, 6 male and 94 female. In Table 1 were reported demographic and SLE disease feature of enrolled sample.

**Table 1:** Demographics and lupus disease features.

Demographic Features	Number, N	100
	Sex, M/F, N (%)	6/94 (6%/94%)
	Age, years, mean ± SD	47.5 ± 14.1
	Disease duration, years, mean ± SD	9.9 ± 7.3
Disease component (according to BILAG: almost 1 BILAG from A to C)	Neuro-Psychiatric disease, N (%)	27 (27)
	Active Renal disease, N (%)	11 (11)
	Costitutional disease, N (%)	72 (72)
	Muscoloskeletal disease, N (%)	40 (40)
	Cardiovascular and respiratory disease, N (%)	16 (16)
	Vasculitis, N (%)	8 (8)
	Mucocutaneous disease, N (%)	43 (43)
	Hematologic disease, N (%)	28 (28)
Previous Involvement (BILAG D)	Previous Renal disease, N (%)	20 (20)
Anti-phospholipid Syndrome		21 (21)
Serologic features	Anti-dsDNA positive, N (%)	47 (47)
	Ipo-complement C3 and/or C4, N (%)	52 (52)
SLE therapy	Mean monthly actual glucocorticoids dosage, mg of prednisone or equivalents, mean ± SD	147.7 ± 127.7
	Ongoing Oral glucocorticoid therapy, N (%)	72 (72)
	Cumulative exposure to glucocorticoids, years, mean ± SD	8.1 ± 7.3
	Cumulative exposure to glucocorticoids, percentage of disease duration, mean ± SD	79% ± 33%
	Cumulative exposure to high dose glucocorticoids (daily dose ≥ 7.5 mg), years, mean ± SD	3.8 ± 5.4
	Cumulative exposure to high dose glucocorticoids (daily dose ≥ 7.5 mg), percentage of disease duration, mean ± SD	40% ± 41%
	Antimalarial, ongoing, N (%)	68 (68)
	Antimalarial, cumulative exposure, years, mean ± SD	5.1 ± 4.3
	Antimalarial, cumulative exposure, percentage of disease duration, mean ± SD	60% ± 38%
	Azathioprine, N (%)	36 (36)
	Methotrexate, N (%)	17 (17)
	Mycophenolate Mofetil, N (%)	14 (14)
	Oral Cyclophosphamide, N (%)	6 (6)
	IV Cyclophosphamide (in the last 1 year), N (%)	6 (6)
	Other immunosuppressant, N (%)	8 (8)
	Rituximab in the last 2 years, N (%)	10 (10)
	Belimumab in the last 2 years, N (%)	0 (0)
Disease Activity and Damage	Actual SELENA-SLEDAI, mean ± SD	3.7 ± 4.1
	Mean SELENA-SLEDAI last year, mean ± SD	4.0 ± 4.8
	Mean number of flares, last 12 months, mean ± SD	0.5 ± 0.9
	SDI, mean ± SD	0.6 ± 0.8

Abbreviations: M, male; F, female; IV, intra-venous; BILAG, British Isles Lupus Assessment Group [41]

### Metabolic Syndrome prevalence and parameters

Results regarding MeS parameters and the other CVDs risk factors in SLE patients were summarized in Table 2. MeS prevalence in our SLE sample was 34%. The prevalence of MeS components was: obesity in 46% of patients, raised triglycerides in 23% and reduced HDL cholesterol in 26%, raised blood pressure in 45% of patients, impaired fasting glucose in 11% of patiens.

**Table 2:** MeS parameters according to IFD definition.

IFD Metabolic Syndrome Parameters	Metabolic Syndrome, N (%)	34 (34)
	Obesity (waist circumference ≥ 80 cm for women and ≥ 94 com for men), N (%)	46 (46)
	Raised triglycerides level ≥ 150 mg/dL or specific treatment for this lipid abnormality, N (%)	23 (23)
	Reduced HDL cholesterol < 50mg/dL in females, or specific treatment for this lipid abnormality, N (%)	26 (26)
	Raised blood pressure systolic BP ≥ 130 or diastolic BP ≥ 85 mm Hg, or treatment of previously diagnosed hypertension, N (%)	45 (45)
	Raised fasting plasma glucose (FPG) ≥ 100 mg/dL or previously diagnosed type 2 diabetes, N (%)	11 (11)
Other CVD risk factors	Current Smokers, N (%)	14 (14)
	CVD personal history, N (%)	11 (11)
	CVD familiar history, N (%)	25 (25)

### Quality of life in SLE patients with and without MeS

Considering SF-36 summary measures, SLE patients with MeS presented lower scores in both Mental Component Summary (MCS) and in Psysical Component Summary (PCS) compared to SLE patients without MeS. Mean values of MCS in patients with MeS and without MeS were 45.2±25.0 vs 56.1±21.1 respectively (p 0.03). Mean PCS score in patients with MeS compared to patients without MeS were 39.7±19.7 vs 52.5±21.5 respectively (p 0.004). We reported in Table 3 the mean values of MCS and PCS adjusted for confounders.

**Table 3:** Least-squares means of SF-36 components in patients with and without MeS.

Parameter	Class	Mean (SE) Model I	p>t	Mean (SE) Model II	p>t	Mean (SE) Model III	p>t
MCS	MeS-	56.1 (2.6)	0.03	58.9 (4.9)	0.01	58.3 (4.6)	0.03
	MeS+	45.2 (4.3)		46.8 (6.0)		46.8 (5.6)	
PCS	MeS-	52.5 (2.6)	0.004	62.3 (4.8)	0.01	61.9 (4.6)	0.008
	MeS+	39.7 (3.4)		50.0 (5.6)		48.7 (5.2)	
PF	MeS-	63.4 (2.9)	0.01	74.0 (5.6)	0.02	72.3 (5.6)	0.08
	MeS+	49.1 (4.5)		60.6 (6.9)		61.2 (6.8)	
RP	MeS-	49.5 (4.0)	0.02	61.6 (7.8)	0.02	60.8 (7.7)	0.06
	MeS+	32.9 (5.5)		44.3 (9.2)		44.9 (8.9)	
BP	MeS-	54.7 (3.3)	0.003	64.1 (5.7)	0.02	64.5 (5.6)	0.003
	MeS+	40.2 (3.4)		51.6 (6.2)		46.9 (5.7)	
GH	MeS-	42.5 (2.1)	0.13	48.9 (3.9)	0.14	48.6 (3.9)	0.15
	MeS+	36.8 (3.1)		42.9 (4.9)		42.3 (4.7)	
VT	MeS-	49.3 (2.3)	0.24	55.9 (4.5)	0.20	55.4 (4.4)	0.07
	MeS+	43.3 (4.5)		49.1 (6.2)		45.8 (5.7)	
SF	MeS- MeS+	56.9 (2.9) 43.7 (4.6)	0.018	60.5 (5.6) 46.6 (6.8)	0.02	58.9 (5.3) 47.3 (6.6)	0.06
RE	MeS-	58.8 (4.6)	0.04	60.0 (8.3)	0.02	60.6 (8.4)	0.02
	MeS+	43.0 (6.3)		40.8 (9.4)		40.1 (9.6)	
MH	MeS-	59.4 (2.5)	0.09	62.1 (4.9)	0.04	60.1 (4.7)	0.30
	MeS+	50.9 (4.4)		51.9 (5.7)		54.6 (5.7)	

Model I: unadjusted. Model II adjusted for sex, age, education level and TAS. Model III: adjusted for disease duration, mean average of last year SELENA SLEDA, last SDI, years of cumulative exposure to high dose GH and years of exposure to antimalarials., education level, TAS. Abbreviations: SE, standard error. MeS+, patients with MeS. MeS-, patients without MeS.

We reported in Table 3 the mean values of Physical Functioning (PF), Role Physical (RP), Bodily Pain (BP), General Health Perception (GH), Vitality (VT), Social Functioning (SF), Role Emotional (RE) and General Mental Health (MH) in subjects with and without MeS.

### SLE disease features and QoL related factors in patients with and without MeS

Significant differences were not found in disease duration and cumulative exposure to glucocorticoids (GC) among SLE patients with and without MeS diagnosis. Compared to patients without MeS, SLE patients with MeS diagnosis presented an higher age, elevated mean recent SLEDAI score (expressed as an average of the last year disease activity), higher number of organ damage (SDI), higher number of disease flares, and a greater exposure to high doses of GC (daily GC doses ≥ 7.5 mg of prednisone or equivalents. Moreover, we found a lower cumulative exposure to antimlarials in patients with MeS compared to patients without MeS. SLE patients with MeS presented a significantly raised score of depressive symptoms (BDI) and anxiety symptoms (HAM-H). These results were confirmed in last square means comparison using mixed regression models adjusted for age, education levels and alexithymia score (TAS). Moreover, we found a reduced score of physical activity (IPAQ) in patients with MeS. Significant differences were not found in fatigue, sleep disorders score and alexithymia score among SLE patients with and without MeS diagnosis. We reported detailed results in Table 4.

**Table 4:** Least-squares means of SLE-related variables and QoL-related variables in patients with and without MeS.

Parameter	Class	Mean (SE) Model I	p>t	Mean (SE) Model II	p>t	Mean (SE) Model III	p>t
Disease related parameters							
Age, years	MeS-	44.7 (1.8)	0.003	47.6 (3.0)	0.003*		
	MeS+	52.9 (2.0)		55.7 (3.2)			
Disease Duration, years	MeS-	9.2 (0.8)	0.2	9.5 (1.5)	0.47**		
	MeS+	11.2 (1.4)		10.7 (1.9)			
Actual SLEDAI	MeS-	2.9 (0.4)	<0.000 1	2.3 (0.8)	0.001**		
	MeS+	7.6 (0.9)		5.6 (1.1)			
Number of flares, last year	MeS-	0.29 (0.09)	0.01	0.16 (0.17)	0.012**		
	MeS+	0.79 (0.19)		0.7 (0.25)			
Actual SDI	MeS-	0.36 (0.09)	<0.0001	0.3 (0.2)	0.0005**		
	MeS+	1.12 (0.1)		0.9 (0.2)			
Cumulative exposure to GC, years	MeS-	7.1 (1.5)	0.09	6.9 (1.5)	0.17**		
	MeS+	10.0 (0.8)		9.4 (2.1)			
Cumulative exposure to GCs, %	MeS-	0.75 (0.04)	0.051	0.7 (0.07)	0.012**		
	MeS+	0.88 (0.04)		0.9 (0.08)			
Cumulative exposure to hd GC, years	MeS-	2.4 (0.6)	0.001	3.1 (1.1)	0.001**		
	MeS+	6.4 (1.0)		7.6 (1.5)			
Cumulative exposure to hd GC, %	MeS-	0.24 (0.04)	<0.000 1	0.25 (0.07)	<0.0001**		
	MeS+	0.69 (0.05)		0.76 (0.09)			
Cumulative exposure to antimalarial, years	MeS-	5.7 (0.5)	0.023	5.2 (0.9)	0.01**		
	MeS+	3.8 (0.6)		2.8 (1.1)			
Cumulative exposure to antimalarial, %	MeS-	0.74 (0.04)	<0.000 1	0.63 (0.07)	<0.0001**		
	MeS+	0.41 (0.06)		0.29 (0.09)			
QoL related parameters							
BDI score	MeS-	9.1 (0.9)	0.004	6.9 (1.6)	0.01	6.9 (1.6)	0.02
	MeS+	15.5 (1.9)		12.9 (2.5)		12.1 (1.6)	
HAM score	MeS-	11.9 (1.0)	0.02	10.3 (1.8)	0.03	9.4 (1.9)	0.01
	MeS+	17.7 (2.1)		15.5 (2.7)		15.6 (2.7)	
IPAQ score	MeS-	2.3 (0.1)	0.001	2.5 (0.2)	0.01		
	MeS+	1.6 (0.1)		1.9 (0.2)			
Facit-Fatigue score	MeS-	34.6 (1.2)	0.12	38.4 (2.2)	0.38	38.7 (2.3)	0.44
	MeS+	30.7 (2.1)		36.1 (3.0)		36.5 (2.3)	
PSQI score	MeS-	6.5 (0.5)	0.12	6.7 (0.8)	0.22		
	MeS+	8.1 (0.9)		8.1 (1.2)			
TAS-20 score	MeS-	48.6 (1.9)	0.44	50.3 (3.4)	0.75		
	MeS+	51.4 (3.1)		51.6 (4.6)			

Model I: unadjusted. Model II adjusted for sex, age, education level and TAS. Model III: adjusted for disease duration, mean average of last year SELENA SLEDA, last SDI, years of cumulative exposure to high dose GC and years of exposure to antimalarials, education levels and TAS.

*Model II adjusted for sex.

**Model II adjusted for sex, age. Legend: SE, standard error. MeS+, patients with MeS. MeS-, patients without MeS. GC, glucocorticoids. hd GC, high doses of glucocorticoids (daily dose ≥ 7.5 mg of prednisone or equivalents). %, exposure time expressed as percentage of disease duration.

A greater proportion of patients with MeS were seropositive (low complement levels and/or anti-dsDNA positivity), presented low complement levels and presented active renal disease (almost renal BILAG C). 21 of 34 patients with MeS (67.6%) presented low education level (primary or lower secondary school). Conversely, 45 of 66 patients without MeS (68.2%) presented education levels almost equal to upper secondary school (Table 5).

**Table 5:** Analysis categorical variable SLE-related and QoL–related in patients with and without MeS.

Factor	MeS- (N 66)	MeS+ (N 34)	P
*Sex*, *N (%)*			
Male	4 (6.1)	2 (5.9)	0.97
Female	62 (93.9)	32 (94.1)	
***SLE related factors***			
Seropositive, N (%)	34 (51.5)	26 (76.5)	0.01
Low complement levels, N (%)	29 (43.9)	23 (67.6)	0.02
Neuro-Psychitric disease, N (%)	11 (16.7)	16 (47.1)	0.001
Active Renal disease, N (%)	3 (4.5)	8 (23.5)	0.004
***QoL related factors***			
*Education level*			
None	0 (0)	0 (0)	0.03
Primary education	0 (0)	2 (5.9)	
Lower Secondary education	21 (31.8)	21 (61.7)	
Upper Secondary education	26 (39.4)	8 (23.6)	
University degree or upper education levels	19 (28.8)	3 (8.8)	
Physically inactive (IPAQ = 1)	14 (21.2)	19 (55.8)	0.001

Legend. Seropositive: ANA (Anti-nuclear antibodies) positivity + anti-dsDNA positivity and/or ipo-complement.

### Variables associated to SF-36 summary components

A multinomial logistic regression model was used to analyze variables associated to the odds ratio to be in the upper quartiles of MCS and PCS values distribution. The lowest quartile of MCS and PCS values were used as reference. In the univariable analysis, the variables that reduce the odds ratio of being in the upper quartile of MCS were higher education level, the average of recent SLEDAI values, the diagnosis of MeS, the BDI and HAM-H scores, the PSQI score, the TAS-20 score and to be physically inactive. The variable associated to odds ration below 1 of being in the upper quartile of PCS were the same as observed for MCS, plus age, female sex, cumulative years of exposure to high dose GC, the SDI score and the number of recent disease flares. MeS was significantly associated to a reduced odds ratio to be in the upper quartile of MCS and PCS also in multivariable models, after adjustment for demographic and disease related variables. Nevertheless, the significant association between MeS and the odds ratio of being in the upper MCS and PCS quartile was lost when the model was adjusted for BDI score, PSQI score, TAS-20 score and the state of physical inactivity. The detailed results were reported in Table 6.

**Table 6:** Multinomial regression model (dependent variable: quartiles of MCS and PCS values; upper quartile vs lower quartiles). Analysis of variables associated to MCS and PCS in SLE.

Indipendent Variable	Dependent Variable (multinomial distribution: upper quartile vs others)	Univariable		Multivariable Model I		Multivariable Model II	
		OR (95% CI)	p	OR (95% CI)	p	OR (95% CI)	p
Age, years	MCS	0.98 (0.96–1.01)	0.19				
	PCS	0.97 (0.94–0.99)	0.02	0.97 (0.94/0.99)	0.02	0.97 (0.94/1.02)	0.14
Education Level (≥upper secondary vs ≤lower secondary)	MCS	0.41 (0.19–0.8)	0.02	0.25 (0.11/0.57)	0.0008	0.22 (0.09/0.56)	0.001
	PCS	0.76 (0.37–1.53)	0.44	0.39 (0.16/0.98)	0.04	0.35 (0.13/0.96)	0.04
Sex (female vs male)	MCS	0.70 (0.16/3.1)	0.64				
	PCS	0.17 (0.03/0.88)	0.04	0.14 (0.02/0.85)	0.03	0.44 (0.05/3-63)	0.45
Disease Duration, years	MCS	1.01 (0.97/1.07)	0.54				
	PCS	1.01 (0.96/1.06)	0.76				
Low complement (yes vs no)	MCS	1.09 (0.54/2.19)	0.81				
	PCS	0.79 (0.39/1.59)	0.49				
Anti-phospholipid syndrome (yes vs no)	MCS	1.42 (0.60/3.38)	0.42				
	PCS	0.64 (0.27/1.53)	0.30				
Cumulative exposure to hd GC, years	MCS	0.96 (0.89/1.03)	0.23				
	PCS	0.93 (0.86/0.99)	0.04	0.94 (0.86/1.03)	0.17	0.89 (0.80/1.02)	0.05
Cumulative exposure to antimalarials, years	MCS	1.05 (0.96/1.14)	0.26				
	PCS	1.03 (0.95/1.12)	0.48				
Mean SLEDAI last year	MCS	0.90 (0.82/0.99)	0.03	0.89 (0.81/0.99)	0.04	0.96 (0.86/1.08)	0.53
	PCS	0.87 (0.79/0.96)	0.006	0.90 (0.79/1.02)	0.09	0.94 (0.81/10.08)	0.36
Actual SDI	MCS	0.84 (0.56/1.25)	0.38				
	PCS	0.56 (0.36/0.86)	0.008				
Number of disease flares last year	MCS	0.85 (0.57/1.26)	0.42				
	PCS	0.6 (0.39/0.93)	0.03	0.85 (0.49/1.47)	0.56	0.91 (0.52/1.61)	0.75
Physically inactive (IPAQ = 1) (yes vs no)	MCS	0.40 (0.18/0.90)	0.03			0.95 (0.34/2.65)	0.91
	PCS	0.19 (0.08/0.44)	0.0001			0.41 (0.14/1.22)	0.11
BDI score	MCS	0.85 (0.80/0.90)	<0.0001			0.89 (0.82/0.97)	0.009
	PCS	0.83 (0.78/0.88)	<0.0001			0.87 (0.79/0.95)	0.004
HAM score	MCS	0.86 (0.81/0.90)	<0.0001				
	PCS	0.84 (0.79/0.89)	<0.0001				
PSQI	MCS	0.85 (0.77/0.94)	0.001			0.99 (0.87/1.14)	0.95
	PCS	0.80 (0.72/0.89)	<0.0001			0.94 (0.81/1.09)	0.42
TAS-20 score	MCS	0.95 (0.93/0.97)	<0.0001			0.97 (0.93/1.00)	0.08
	PCS	0.97 (0.94/0.99)	0.005			1.02 (0.99/1.07)	0.19
Metabolic Syndrome	MCS	0.42 (0.19/0.90)	0.03	0.39 (0.16/0.92)	0.03	0.62 (0.22/1.73)	0.36
	PCS	0.30 (0.14/0.66)	0.003	0.36 (0.13/0.97)	0.04	0.56 (0.18/1.70)	0.30

Multivariable Model I: adjusted for demographic and SLE disease variables (only variables with significant results in univariable analysis were included). Multivariable Model II: adjusted for Model I variables + QoL related variables (depressive symptoms, alexithymia, physical activity)

### SLE-related variables, QoL-related variables and probability of MeS

In Table 7 we reported the results of univariable and multivariable logistic regression having as depend binary variable the diagnosis of MeS. In the final model, we found that the increase of years of exposure to high doses of glucocorticoids, the average of recent disease activity (mean SLEDAI of the last year) and the increase in the score of depressive symptoms (BDI) are positively associated to the probability of having MeS. Conversely, the increase of years of exposure to antimalarials reduced the probability of having MeS.

**Table 7:** Multiple logistic regression analysis investigating the effects of SLE-related parameters and QoL-related parameters on MeS.

Dependent Variable: MeS Event: having MeS diagnosis	Univariable Logistic Regression		Multivariable Logistic Regression	
Variable	OR (95% CI)	p	OR (95% CI)	p
Age, years	1.04 (1.01/1.08)	0.07		
Education Level (≥upper secondary vs ≤lower secondary)	0.3 (0.1/0.7)	0.005		
Sex (male vs female)	0.97 (0.17/5.57)	0.9		
Disease Duration, yeasr	1.04 (0.98/1.1)	0.2		
Low complement (yes vs no)	2.7 (1.1/6.3)	0.02		
Anti-phospholipid syndrome (yes vs no)	2.7 (1.1/7.2)	0.04		
Active Lupus Nephritis (yes vs no)	6.1 (1.6/26.3)	0.009		
Cumulative exposure to hd GC, years	1.2 (1.1/1.3)	0.002	1.18 (1.06/1.3)	0.004
Cumulative exposure to antimalarials, years	0.8 (0.7/0.9)	0.03	0.82 (0.68/0.98)	0.03
Mean SLEDAI last year	1.2 (1.1/1.4)	0.0003	1.15 (1.0/1.3)	0.04
Actual SDI	2.8 (1.6/4.9)	0.0003		
Number of disease flares last year	1.9 (1.1/3.1)	0.01		
Physically inactive (IPAQ = 1) (yes vs no)	4.4 (1.7/10.9)	0.002		
BDI score	1.1 (1.03/1.15)	0.002	1.1 (1.03/1.17)	0.005
HAM score	1.06 (1.02/1.11)	0.009		
Facit Fatigue score	0.97 (0.9/1.01)	0.1		
TAS score	1.01 (0.9/1.04)	0.4		
PCS score	0.97 (0.94/0.99)	0.007		
MCS score	0.98 (0.96/0.99)	0.03		

## Discussion

Approximately one in three patients enrolled in this study had a diagnosis of MeS according to IFD criteria. This finding is in agreement with the prevalence of MeS described in the scientific literature, considering the variability due to different classification criteria used [20–26]. The high MeS prevalence we have observed in our sample is a consequence of the cross sectional design of this study, allowing the enrollment of patients with a wide range of disease duration, disease activity and therapy exposure, in particular glucocorticoids. Considering these aspects, we underline that the mean age of patients enrolled was about 47 years old and the mean disease duration was about 10 years. Moreover, we observed that the mean proportion of disease duration spent with high doses of glucocorticoids (defined as daily dose ≥ 7.5 mg of prednisone or equivalents) was about 40%.

As previously reported [24–26], we found that, compared with patients without MeS, SLE patients with MeS diagnosis presented older age, higher disease activity, an increased number of recent disease flare and an higher score of organ damage. As demonstrated in general population, also SLE patients with MeS seem to present a low education levels [9].

The first finding of this study was that patients with diagnosis of MeS presented an impoverishment of QoL both in physical and mental summary components of SF-36. We applied a mixed regression model to compare the last square means of SF-36 parameters between subjects with and without MeS, allowing the adjustment for factors with considerable impact on physical and mental health perception as age, sex, education levels. We also decided to include in the models the alexithymia score with the aim of considering also the possible effects on SF-36 results of the impairment of the emotion perception / expression [42].

We evaluated QoL using SF-36 instrument, allowing us to analyze the single QoL components. After adjustment for age, sex, education level and alexithymia, we found a reduction in all individual SF-36 score in patients with MeS, with the exception of vitality and global health. We further evaluated the results of vitality score using Facit-fatigue index. In analogy with what we observed for vitally, patients with and without MeS did not differ for fatigue level. This result was confirmed after correction for age, sex, education level and alexithymia score. We do not have an explanation that the MeS has no impact on the fatigue in our cohort of SLE patients. In SLE patients, fatigue was reported to be strongly associated to mood disorders and, in several study, to sleep quality and stress, while the relation of fatigue with disease activity and damage was controversial [43]. In general population, the impact of MeS on fatigue is still poorly explored [12]. None of the available study on SLE patients reported a relation between fatigue and MeS or MeS components, such as obesity. We propose to investigate this aspect in the prospective extension of this study.

Since we observed an increase of disease activity, damage index and exposure to therapy in SLE patients with MeS compared to those without MeS, we decided to adjust SF-36 components also for average of recent SLEDAI, SDI score, cumulative years of high dose GC therapy and antimalarial therapy. Summary component of SF-36 were reduced in MeS also after controlling for SLE disease features. However, considering the individual SF-36 component after control for SLE disease feature, only bodily pain and role emotional remained significantly different between patients with and without MeS.

To deepen the impact of MeS on QoL of SLE patients, we built a multinomial logistic regression model having as dependent variable the MCS or PCS quartiles. As extensively reviewed elsewhere [43], we found that age, the average of recent disease activity, the organ damage score and the number of recent flares reduced the probability of have high values of PCS. Moreover, we observed that the odds ratio of been in the upper PCS quartile was reduced by the years of cumulative exposure to high dose GC. In accordance with what has already been described, the distribution of MCS values is more arduous to describe with a statistical model. Interestingly, we observed that higher education levels reduced the probability of having better QoL, both in mental and physical components. This phenomenon has already been described in middle-aged woman of general population [44]. As previously reported, in our analysis, the distribution of MCS and PCS values was strongly associated with the severity of symptoms of mood disorders, the sleep quality score, the physical activity and the score of alexithymia construct [43]. In our analysis, MeS was inversely related to the probability of being in the higher MCS and PCS quartiles even adjusted for demographic and disease features as age, education level, average of recent SLEDAI, cumulative exposure to high dose GC. This significant association of MeS and QoL was lost when the model was enriched with QoL related variables evaluating mood disorder symptoms, sleep quality, physical activity and alexithymia. This observation suggests that much of the MeS impact on QoL may be mediated by mood disorders and, to a lesser extent, by poor physical activity.

We evaluated the extent of symptoms of mood disorders in our SLE sample. In particular, we explored depressive and anxiety symptoms. According to our findings, SLE patients meeting MeS criteria are burdened by depressive and anxiety symptoms more seriously than patients without MeS. The scores evaluating the extent of manifestations related to mood disorders remain high in MeS also after control for confounders as age, sex, education levels and alexithymia. The relation of mood disorders with MeS and MeS components, in particular obesity, was widely demonstrated. A recent meta-analysis on 29 cross-sectional studies and 11 cohort studies suggested that it could exist a bidirectional association of MeS and depression [11]. Several cross sectional studies and a meta-analysis on 15 cohort studies seem demonstrate a reciprocal link between depression and obesity. The conclusion of the meta-analysis was that obesity could increase the risk of depression, and depression was found to be predictive of developing obesity [45]. The pathophysiological basis of these relation between MeS with reduced QoL remain to be elucidated in SLE patients as well as in general population. Several explanations have been proposed. Firstly, the MeS is often accompanied by CVDs and type 2 diabetes, as end-organ damage, consequences of MeS. Both of these conditions are associated with a reduced QoL [46, 47]. Moreover, both MeS and obesity are characterized by the activation of several inflammatory pathways, including cytokines as Interleukin-6, Tumor Necrosis Factor-alpha, Interleukin-1 and adipokines as leptin, adiponectin and resistin [13, 48, 49]. These inflammatory mediators have been demonstrated to be involved in depression and anxiety [50]. Another possible pahopshysiological mechanism could involve the hypothalamic-pituitary-adrenal axis (HPA axis). In particular, insulin-resistance and weight gain, both associated to MeS, lead to HPA-axis dysregulation [51]. An implication of HPA-axis dysregulation in depression has been widely demonstrated [52]. Indeed, it needs to be considered also the role of psychological distress related to being overweight. Body image alteration and satisfaction in obese patients could be a potential mediator of the relationship between obesity and psychological distress [53]. Finally, we must consider the role of reduced physical activity in lowering the QoL in patients with MeS as well as in obese subjects [54, 55]. In SLE patients, Pinto et al recently reported a reduced QoL in all SF-36 domains in a cross section study on 21 physically inactive patients [56]. We found that a larger proportion of SLE patients with MeS are physically inactive according to IPAQ score, while patients without MeS are frequently phy sically active and sometimes meet HEPA criteria for adequate physical activity [38].

To further evaluate the relation between MeS and QoL related factors, we build a logistic regression model using as predicted event the diagnosis of MeS. As we expected, several SLE related factor increase the odds ratio of MeS in SLE patients, such as the low complement status, the diagnosis of anti-phospholipid syndrome, the presence of active lupus nephritis, the average values of recent disease activity expressed by SELENA-SLEDAI, the number of disease flares in the last year and the time of exposure to high doses of glucocorticoids. Furthermore, our results underline the positive impact on MeS odds ratio of QoL related variables such as to be physically inactive, the values of the BDI and HAM-H scores and the values of SF-36 summary physical and mental components. In the final multivariable models, we found that the odds ratio of MeS in our SLE sample was associated to the SELENA-SLEDAI score, to the extent of depressive symptoms and to the duration of exposure to high doses of glucocorticoids. Interestingly, the length of therapy with antimalarials seems to exert a protective role on the probability of having MeS, according to out results.

We observed that the extent of antimalarial therapy could reduce the odds ratio of MeS. We recently demonstrated that hydroxychloroquine assumed for more than five years protects against the first cardiovascular event in a large retrospective lupus cohort [57]. Beside the well-known effects on SLE disease, as the reduction of flares risk, the steroid sparing effect, the reduction of organ damage accrual and the prevention of the thrombotic effects of anti-phospholipid antibodies [58, 59], a crescent body of evidences supports the beneficial impacts of antimalarial on cardio-metabolic diseases including diabetes mellitus and dyslipidemia. The biological mechanism of these positive effects has not yet been clarified but may include alterations in insulin metabolism and signaling through cellular receptors [60].

Our study presents several limitations. First of all, the cross sectional design of the study does not allow to analyze a causative relation between MeS, QoL and related factors. Moreover, the sample size, and consequently the number of patients with diagnosis of MeS, reduces the possibility to include other variables in the final multivariable regression model. Moreover, the sample size limits the possibility of stratifying the analysis according to therapy exposure. One of the strengths of our work is the evaluation of multiple dimensions related to QoL (mood disorders, fatigue, sleep quality, alexithymia). Another one is the use of mixed models for comparing the values of SF36, which has allowed to consider possible confounders.

In conclusion, according to our findings, MeS seems to be associated with an impoverishment of QoL in SLE patients, both in mental and physical components. Moreover, SLE patients with MeS presented an increased amount of symptoms of mood disorders and are often physically inactive.

MeS is associated to a reduction of the probability of having values of MCS and PCS in their upper quartiles of distribution, even after adjustment for confounders regarding demographic and SLE disease features. Conversely, when we add to the model the scores of the mood disorders symptoms, sleep quality, physical activity and alexithymia, MeS losses its significant impact on MCS and PCS values. This observation underlines the central role of mood disorders and physical activity in the impact of MeS on QoL measures.

The factors mainly associated to the presence of MeS in SLE were the recent disease activity, the duration of exposure to high dose glucocorticoids and the severity of depression, while the length of therapy with antimalarials seems to except a protective action.

Overall, these evidences underline the need to maximize the control of disease activity, minimizing as much as possible the use of high doses of glucocorticoids and potentially using antimalarials in all SLE patients, in order to manage the cardio-metabolic risk. Alongside these aspects, we have to increase our awareness of the need to properly manage mood disorders in these patients. In particular, it seems necessary to put in place all pharmacological and non-pharmacological measures to try to improve the quality of life in lupus patients with MeS.
